# Associations of Obstructive Sleep Apnea Risk with Obesity, Body Composition and Metabolic Abnormalities in School-Aged Children and Adolescents

**DOI:** 10.3390/nu16152419

**Published:** 2024-07-25

**Authors:** Zijun Liao, Yiren Chen, Lijun Wu, Yiying Huang, Shaoli Li, Junting Liu, Xinnan Zong, Jun Tai, Fangfang Chen

**Affiliations:** Capital Institute of Pediatrics, Beijing 100020, China; zijun_liao@yeah.net (Z.L.); yirenchen203@163.com (Y.C.); wulijun19801110@126.com (L.W.); hyyhyy2016@163.com (Y.H.); shaolichina1@163.com (S.L.); winnerljt@163.com (J.L.); xnzong@sina.com (X.Z.)

**Keywords:** obstructive sleep apnea, childhood obesity, body composition, metabolic health

## Abstract

The objective of this study is to explore the associations between obesity, body composition, and the self-reported risk of obstructive sleep apnea (OSA) and to examine whether the risk of OSA is related to metabolic abnormalities in children and adolescents aged 6–17 years. Utilizing data from the 2022 to 2023 Beijing Children and Adolescents Health Cohort baseline survey, 5000 school-aged participants were analyzed. OSA risk was assessed via the Pediatric Sleep Questionnaire, with anthropometric and body composition measurements taken. Metabolic markers included blood pressure, lipid levels, blood glucose, and uric acid. Associations were analyzed using logistic regression and generalized linear models. Results showed that 88.6% were low-risk and 11.4% were high-risk for OSA. Overweight (aOR 1.53, 95% CI 1.22–1.92), obesity (aOR 1.94, 95% CI 1.57–2.40), and abdominal obesity (aOR 1.59, 95% CI 1.31–1.93) significantly increased OSA risk. High fat mass was a critical factor, while muscle mass was not, especially in those who were overweight and obese. Associations of OSA risk with metabolic abnormalities were non-significant after adjusting for BMI. Our research highlights the significant associations of obesity and body composition with OSA risk, with child BMI influencing the relationship between OSA and metabolic abnormalities. Future research should explore causative relationships and the enduring impacts of OSA on metabolic health in children.

## 1. Introduction

Obstructive sleep apnea (OSA) is a condition defined by the frequent occurrence of either a total or partial collapse of the upper respiratory tract, resulting in intermittent oxygen deprivation and hypercapnia [1]. The rate of OSA is on the rise and is prevalent among children [2]. Untreated OSA is linked to negative health outcomes that include behavioral problems, decreased neurocognitive development, and metabolic abnormalities [2]. 

Childhood obesity, which has also been on the rise globally, is a risk factor for pediatric OSA; excessive fat deposition in the throat and neck, together with larger adenoids and tonsils, primarily contributes to OSA [3,4,5,6]. For school-aged children and adolescents, obesity may play a more substantial role than adenotonsillar hypertrophy [7]. Research in adults has shown that sarcopenic obesity, rather than obesity alone, is associated with OSA, indicating a complex connection between unhealthy body composition and OSA [8]. In obese children, the neck-to-abdominal fat percentage is also significant in the development of OSA [9]. However, the impacts of comprehensive body composition on OSA, especially in normal-weight children, remain unclear. Understanding the correlations involving obesity, body composition, and OSA risk in children and adolescents is critical for developing effective interventions.

OSA impacts metabolic markers such as blood pressure and lipids, but the results are inconsistent regarding whether OSA exerts an effect independent of obesity [6,10,11,12,13,14,15]. Most research has focused on a limited range of markers, making it challenging to comprehensively assess the impacts of OSA on metabolic health. This study considers weight status when exploring the associations between OSA and metabolic markers, investigating a complete spectrum of metabolic markers including blood pressure, lipid levels, blood glucose, and uric acid. 

Polysomnography (PSG) is considered the gold standard for OSA diagnosis [16]. Nevertheless, the available resources frequently prove insufficient to manage the substantial influx of referrals for testing, leading to long wait times for pediatric PSG [17]. Furthermore, the cost of PSG makes it unlikely to be used as a monitoring tool. Therefore, it is crucial to identify children who are at risk for OSA based on obesity and body composition and to understand the negative changes in children with high risk of OSA to prioritize PSG testing effectively.

The objective of this study is to examine whether obesity and body composition are associated with self-reported risk for OSA and whether the risk for OSA is related to metabolic abnormalities in children and adolescents between the ages of 6 and 17.

## 2. Materials and Methods

This report adhered to the reporting guidelines of the Strengthening the Reporting of Observational Studies in Epidemiology (STROBE) statement [18].

### 2.1. Study Design and Participants

The data for this study were obtained from the baseline survey in the Beijing Children and Adolescents Health Cohort conducted from 2022 to 2023, which was a prospective cohort study design established in a district of Beijing, China. The cohort consisted of 11 representative kindergartens and primary and secondary schools, including a total of 6013 participants aged 3–17 years. The study was carried out following the Declaration of Helsinki and received approval from the Ethics Committee of the Capital Institute of Pediatrics (SHERLL2022043).

### 2.2. Data Collection and Measurements

#### 2.2.1. Sleep Assessment

The parents of pediatric participants completed the questionnaires, with the child’s help if necessary. The Pediatric Sleep Questionnaire (PSQ) was a recommended screening questionnaire in the American Academy of Pediatrics OSA guidelines, and the validated simplified Chinese version of PSQ was used to assess OSA risk [19,20,21]. The PSQ comprises 22 yes/no items, with “yes” scored as 1 and “no” scored as 0; a total score of 7 or higher is considered indicative of high risk for OSA [19,20]. 

#### 2.2.2. Anthropometric Measurements

The height of the children was measured without shoes using a mechanical stadiometer (Harpenden Portable Stadiometer, Crymych, Dyfed, UK) by trained staff. After a calm expiration, the waist circumference was measured horizontally around the navel. The body composition analyzer (H-Key350, Beijing Seehigher Technology Co., Ltd., Beijing, China) using segmental multi-frequency bioelectrical impedance analysis (BIA) was employed to measure body weight and body composition, including percentage of fat mass (FM%), fat mass, fat-free mass, total muscle mass, and trunk fat mass [22,23]. Total muscle mass is estimated based on measurements of fat-free mass in the trunk and limbs. Children must be fasting and have an empty bladder prior to measurements. The formulas for calculating body mass index (BMI), fat mass index (FMI), fat-free mass index (FFMI), and total muscle mass index (TMMI) were the corresponding mass in kilograms divided by the square of height in meters, respectively [22]. The central fat mass index (CFMI) was calculated as trunk fat mass in kilograms divided by the square of height in meters [24]. The fat-to-muscle mass ratio (FMR) was computed as total body fat mass divided by muscle mass [25]. 

Based on the WHO growth standards, BMI z score (zBMI) was calculated age- and sex-standardized [26,27,28], and children with zBMI above +1 SD were classified as overweight, while those with zBMI above +2 SD were classified as obese, respectively [27,29,30]. Abdominal obesity was defined by a waist-to-height ratio (WHtR) equal to or greater than 0.5 [31,32,33]. The FMI and TMMI were also age- and sex-standardized. Body composition phenotypes were derived based on whether FMI and TMMI z scores were ≥1, resulting in four phenotypes: normal fat-normal muscle mass, normal fat-high muscle mass, high fat-normal muscle mass, and high fat-high muscle mass [34].

#### 2.2.3. Metabolic Indicators

Blood pressure in mmHg, including systolic blood pressure (SBP) and diastolic blood pressure (DBP), was measured three times using an oscillometric sphygmomanometer (HBP-1300, Omron, Kyoto, Japan) on the right upper arm. Venous blood (5 mL) was collected after 12 h of fasting. The levels of serum total cholesterol (TC), triglycerides (TG), HDL-cholesterol (HDL-C), LDL-cholesterol (LDL-C), uric acid (UA), and fasting plasma glucose (FPG) were measured by a Siemens Automatic Biochemistry Analyzer (ADVIA 2400, Siemens, Erlangen, Germany). 

Metabolic abnormalities were defined as follows: hypertension was defined as an SBP and/or DBP that was equal to or greater than the 95th percentile for individuals of the same sex, age, and height percentile population [35,36]. High TC levels were defined as TC concentration equal to or greater than 5.17 mmol/L; high TG levels were defined as TG equal to or greater than 1.12 mmol/L for children aged ≤9 years and 1.46 mmol/L for those aged ≥10 years; low HDL-C levels were defined as HDL-C concentration less than 1.03 mmol/L; and high LDL-C levels were defined as LDL-C concentration equal to or greater than 3.36 mmol/L [37]. Impaired fasting glucose (IFG) was defined as FPG equal to or greater than 5.6 mmol/L [38]. High UA levels were defined as UA concentrations greater than 420 mmol/L for boys and 360 mmol/L for girls [39]. 

#### 2.2.4. Covariates

The study collected data on various potential covariates using questionnaires. These covariates included maternal age at delivery, maternal history of gestational diabetes mellitus/hypertension, delivery mode, child sex, birthweight, exclusive breastfeeding duration in the first six months, family history of metabolic abnormalities, annual family income, maternal education, paternal education, maternal current BMI, paternal current BMI, child age, sugary beverage consumption pattern, and passive smoking in the past week.

### 2.3. Statistical Analyses

Differences between groups of OSA risk were compared using the *t*-test for means, the Kruskal–Wallis test for medians, and the chi-square test for frequencies.

Associations of obesity, abdominal obesity, and body composition phenotypes with OSA risk were evaluated using logistic regression models. Multivariate models adjusted for maternal age at delivery (<35, ≥35 years), maternal history of gestational diabetes mellitus/hypertension (without, with), delivery mode (vaginal delivery, cesarean delivery), child sex (male, female), exclusive breastfeeding duration in the first six months (yes, no), family history of metabolic abnormalities (without, with), annual family income (<20,000, 20,000–120,000, 120,000–250,000, ≥250,000 CNY), maternal education (high school or less, college, above college), paternal education (high school or less, college, above college), child age, sugary beverage consumption pattern (≥1/day, 4–6/week, 1–3/week, ≤3/month), and passive smoking in the past week (yes, no). The odds ratio (OR) and 95% confidence interval (CI) for OSA risk per unit increase in anthropometric and body composition indicators (i.e., zBMI, FM%, FMI, FFMI, TMMI, CFMI, and FMR) were also examined. 

Associations of OSA risk with metabolic abnormalities were explored using logistic regressions, and associations of OSA risk with metabolic indicators in children were further explored using generalized linear models. In the adjusted analyses, the same aforementioned covariates were adjusted in model 1, and the child BMI was further adjusted in model 2. In sensitivity analyses, child FMI was adjusted in model 2. Subgroup analyses were conducted to investigate the correlations by the child BMI (normal BMI, overweight/obesity) and sex (male, female). Missing values of a covariate were regarded as a category in adjusted analyses. 

A 2-tailed *p* value was deemed statistically significant at <0.05. The statistical analyses were conducted using SAS 9.4 (SAS Institute Inc., Cary, NC, USA).

## 3. Results

Among the 6013 children and adolescents who were investigated, 181 preschool children were excluded, and 832 were further excluded due to missing data regarding PSQ. Consequently, the final analysis included 5000 school-aged children and adolescents. Of these, 4428 (88.6%) were categorized as low-risk for OSA, whereas 572 (11.4%) were classified as high-risk. Table 1 presents the characteristics of the participants according to the risk of OSA. Compared to children with a low risk of OSA, those at high risk were more likely to be male (60.0% vs. 49.5%), have a family history of metabolic abnormalities (71.5% vs. 66.4%), experience passive smoking in the past week (37.1% vs. 26.6%), consume sugary beverages daily (7.3% vs. 6.8%), and have lower proportions of exclusive breastfeeding in the first six months (55.2% vs. 59.7%), normal weight (43.6% vs. 58.7%), and normal fat-normal muscle mass (69.7% vs. 78.4%).

As shown in Table 2, after covariate adjustments, the associations of overweight and obesity with high OSA risk were significant. The adjusted OR (aOR) for overweight was 1.53 (95% CI 1.22 to 1.92), and for obesity, it was 1.94 (95% CI 1.57 to 2.40). Abdominal obesity also significantly increased the odds of high OSA risk (aOR 1.59, 95% CI 1.31 to 1.93). Children and adolescents in the groups of high fat-normal muscle mass and high fat-high muscle mass were more likely to have high OSA risks compared to those in the normal fat-normal muscle mass group [aORs 1.39 (95% CI 1.03 to 1.88) and 2.05 (95% CI 1.56 to 2.69)], but not for those in the normal fat-high muscle mass group (aOR 1.21, 95% CI 0.84 to 1.73).

**Figure 1:**
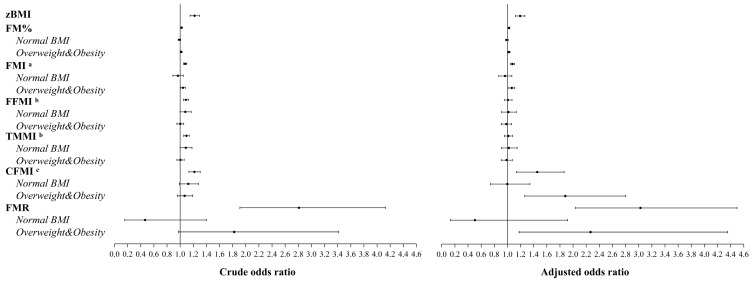
Associations of anthropometric and body composition indicators with OSA risk. Multivariate models were adjusted for maternal age at delivery, maternal history of gestational diabetes mellitus/hypertension, delivery mode, child sex, exclusive breastfeeding duration in the first six months, family history of metabolic abnormalities, annual family income, maternal education, paternal education, child age, sugary beverage consumption pattern, and passive smoking in the past week; ^a^ further adjusted for FFMI; ^b^ further adjusted for FMI; ^c^ further adjusted for TMMI. Abbreviations: OSA, obstructive sleep apnea; zBMI, BMI z score; FMI, fat mass index; FFMI, fat-free mass index; TMMI, total muscle mass index; CFMI, central fat mass index; FMR, fat-to-muscle mass ratio.

For anthropometric and body composition indicators, zBMI (aOR 1.19, 95% CI 1.12 to 1.27), FM% (aOR 1.03, 95% CI 1.02 to 1.03), FMI (aOR 1.08, 95% CI 1.04 to 1.11), CFMI (aOR 1.45, 95% CI 1.13 to 1.86), and FMR (aOR 3.03, 95% CI 2.04 to 4.50) were associated with increased odds of high OSA risk, while FFMI (aOR 1.01, 95% CI 0.95 to 1.07) and TMMI (aOR 1.01, 95% CI 0.95 to 1.08) were not associated with high OSA risk. Subgroup analyses showed that FM% (aOR 1.02, 95% CI 1.01 to 1.04), FMI (aOR 1.07, 95% CI 1.02 to 1.11), CFMI (aOR 1.88, 95% CI 1.26 to 2.80), and FMR (aOR 2.27, 95% CI 1.18 to 4.36) raised the risk of high OSA in overweight and obese children but not in normal-weight children. FFMI and TMMI, on the other hand, had no effect on OSA risk across BMI categories (*p* < 0.05, Figure 1).

In the adjusted model 1 for associations of OSA risk with metabolic abnormalities, children with high OSA risk had higher odds of low HDL-C levels (aOR 1.37, 95% CI 1.01 to 1.88) and high TG levels (aOR 1.34, 95% CI 1.06 to 1.71) compared to those with low OSA risk. In model 2, after adjusting the child BMI, the associations attenuated to non-significance (*p* > 0.05, Table 3). In sensitivity analyses, the associations remained similar after adjustment for child FMI. The associations were not significant in normal-BMI and overweight/obese children (Table S1) nor in boys or girls (Table S2). For continuous metabolic indices, after adjusting for covariates, including the child BMI, most associations were non-significant except for SBP, which was borderline statistically significant but not clinically meaningful (β −0.011, 95% CI −0.018 to −0.004, Table S3).

## 4. Discussion

This study comprehensively examines the associations between OSA risk, obesity, body composition, and metabolic abnormalities in school-aged children and adolescents. Our observations indicate that being overweight, obese, or having abdominal obesity significantly increases the likelihood of high OSA risk. Notably, children and adolescents with high fat mass, regardless of muscle mass, exhibited a significantly higher risk of OSA. Elevated body fat in those overweight and obese contributed to a higher risk of OSA, unlike those of normal weight. Furthermore, the relationships between OSA risk and metabolic abnormalities became non-significant after adjusting for the child BMI.

OSA is the prevailing kind of sleep-disordered breathing and signifies the severe manifestation of sleep-related issues [40]. The worldwide prevalence of childhood obesity, metabolic disorders, and OSA is rising [2,3,41]. For children and adolescents, studies have identified that overweight and obesity are the risk factors of sleep-disordered breathing [5]. Obesity is becoming a significant risk factor for pediatric OSA, particularly among school-aged children and adolescents, despite adenotonsillar hypertrophy being the most common risk factor [42,43]. In this population, our study found that anthropometric measurements can predict OSA, with significant associations between overweight, obesity, and OSA risk. Children who were obese had a higher risk of OSA than those who were overweight. Central adiposity is reported to be a significant risk factor for OSA in adults [44,45]. In children and adolescents, our study also found that abdominal obesity significantly increased the odds of high OSA risk. Our findings align with the prior study recruiting adolescents, showing a positive correlation between higher BMI and increased OSA risk [6].

The links between body composition measured by BIA and OSA have not been fully explored in children and adolescents. In adults, it is reported that OSA screens can use the muscle-to-fat ratio as an indicator in males [46]. Our study underscores the importance of considering comprehensive body composition rather than relying solely on BMI. High fat mass emerged as a crucial factor in elevating OSA risk, while muscle mass alone had no effect. Although muscle mass alone was not a critical factor for OSA risk, in children and adolescents with different body composition phenotypes, those with high fat-high muscle mass had the highest risk of OSA. This could be attributed to the combination of high fat mass and high muscle mass, indicating a generally higher overall body weight and volume, and to the likelihood of ectopic fat accumulation within muscle tissues in children with both high fat mass and high muscle mass [34,47]. Additionally, FMR, which comprehensively reflects body composition balance, markedly increased the OSA risk, indicating that the combined effects of high fat mass and inadequate muscle mass exacerbate the risk of OSA. Stratified analysis showed that higher body fat increased the OSA risk in overweight and obese children and adolescents but not in those who were normal-weight. This suggests that fat accumulation is a primary driver of OSA, emphasizing the need for body composition assessments and the importance of fat mass reduction, especially for obese children. This could be attributed to physiological differences between obese and nonobese airways, such as adipose tissue around the pharynx and neck and more crowding of the oropharynx in obese children [4,48], and accumulation of fat around the viscera and abdominal and thoracic walls, which leads to decreased lung volume and oxygen reserve [13]. 

Studies have linked OSA to a higher likelihood of negative metabolic conditions, with obesity serving as a common comorbidity in individuals with both OSA and metabolic syndrome [15]. Previous studies have tended to focus on one type of indicator, such as blood lipids, blood glucose, or blood pressure, with inconsistent results [10,15,49,50]. It was found that the links between OSA and some metabolic markers (high TG levels and low HDL-C levels) became statistically non-significant when the child BMI was taken into account. The associations were also non-significant after stratifying by the child BMI and sex. This suggests that obesity has a significant influence on the connection between OSA and metabolic problems. The findings align with previous research indicating that the association between OSA and metabolic dysfunction is predominantly determined by obesity rather than OSA [51]. Early or mild OSA may have a negligible effect on metabolic abnormalities; in long-term or severe cases, it may negatively impact the metabolic profile [6,10,52,53,54]. Prospective investigations are required to assess the enduring impact of OSA on metabolic health in children in the future.

Our study had strengths. We comprehensively investigated a wide variety of anthropometric and body composition measurements and metabolic biomarkers, targeting a general population rather than only obese children in many studies. We also used the validated sleep questionnaire to define the risk of OSA. The study also had limitations. The study’s cross-sectional design precluded the determination of causative relationships. Additionally, we did not use PSG to diagnose OSA; given the high demand for limited resources, our objective was to identify children with high OSA risk based on obesity and body composition and to understand negative changes in at-risk children to prioritize PSG testing. We did not exclude children with central sleep apnea (CSA), which may have caused bias in our findings. Future research warrants the use of PSG and the differentiation between OSA and CSA. Future research exploring the associations of segmental muscle mass with OSA risk would be beneficial.

## 5. Conclusions

Our study enhances comprehension of the intricate connections of obesity and body composition with OSA risk in children and adolescents. Elevated fat mass increases the risk of OSA, particularly in obese children. Regarding the associations between OSA risk and metabolic abnormalities, our analyses indicate that these relationships are largely mediated by obesity rather than the OSA risk itself. In the future, it is important to conduct longitudinal studies to examine the causative relationships and enduring effects of OSA on metabolic health in children, thereby guiding better screening and management practices.

## Figures and Tables

**Table 1:** Characteristics according to the risk of OSA.

Characteristics	Low-Risk for OSA (n = 4428)	High-Risk for OSA (n = 572)	*p*
Maternal age at delivery (year), Mean ± SD	27.8 ± 4.3	27.6 ± 4.5	0.267
Maternal age at delivery (year), n (%)			
<35	3917 (93.0)	508 (94.1)	0.368
≥35	296 (7.0)	32 (5.9)	
Maternal history of gestational diabetes mellitus/hypertension, n (%)			
Without	4082 (92.2)	525 (91.8)	0.741
With	346 (7.8)	47 (8.2)	
Delivery mode, n (%)			
Vaginal delivery	1985 (45.9)	268 (48.3)	0.298
Cesarean delivery	2340 (54.1)	287 (51.7)	
Child sex, n (%)			
Male	2194 (49.5)	343 (60.0)	<0.001
Female	2234 (50.5)	229 (40.0)	
Birthweight (g), Mean ± SD	3393.7 ± 659.3	3369.1 ± 592.6	0.426
Birthweight (g), n (%)			
<2500	136 (3.9)	16 (3.8)	0.893
2500–3999	2927 (84.2)	355 (83.5)	
≥4000	415 (11.9)	54 (12.7)	
Exclusive breastfeeding duration in the first six months, n (%)			
yes	2528 (59.7)	302 (55.2)	0.047
no	1703 (40.3)	245 (44.8)	
Family history of metabolic abnormalities, n (%)			
Without	1490 (33.6)	163 (28.5)	0.014
With	2938 (66.4)	409 (71.5)	
Annual family income (CNY), n (%)			
<20,000	760 (18.4)	105 (19.7)	0.708
20,000–120,000	1403 (33.9)	175 (32.9)	
120,000–250,000	1284 (31)	171 (32.1)	
≥250,000	689 (16.7)	81 (15.2)	
Maternal education, n (%)			
High School or less	2371 (55.5)	308 (55.8)	0.749
College	1838 (43)	238 (43.1)	
Above college	64 (1.5)	6 (1.1)	
Paternal education, n (%)			
High School or less	2518 (59.2)	335 (60.4)	0.548
College	1645 (38.7)	205 (36.9)	
Above college	91 (2.1)	15 (2.7)	
Maternal current BMI (kg/m^2^), Mean ± SD	24.1 (5.4)	24.3 (5.5)	0.376
Maternal current BMI (kg/m^2^), n (%)			
<18.5	206 (4.9)	19 (3.5)	0.443
18–23	1843 (44)	231 (43)	
23–27.5	1527 (36.5)	208 (38.7)	
≥27.5	612 (14.6)	79 (14.7)	
Paternal current BMI (kg/m^2^), Mean ± SD	26.3 (5.6)	26.6 (5.2)	0.298
Paternal current BMI (kg/m^2^), n (%)			
<23	916 (22.1)	106 (19.8)	0.006
23–27.5	2011 (48.4)	235 (43.9)	
≥27.5	1227 (29.5)	194 (36.3)	
Child age (year), Mean ± SD	11.0 ± 3.0	11.1 ± 2.9	0.708
Sugary beverage consumption pattern, n (%)			
≥1/day	296 (6.8)	41 (7.3)	0.001
4–6/week	372 (8.6)	58 (10.3)	
1–3/week	1371 (31.7)	217 (38.4)	
<1/week	2291 (52.9)	249 (44.1)	
Passive smoking in the past week, n (%)			
no	3141 (73.4)	349 (62.9)	<0.001
yes	1137 (26.6)	206 (37.1)	
Child weight status, n (%)			
Normal	2592 (58.7)	249 (43.6)	<0.001
Overweight	869 (19.7)	131 (22.9)	
Obesity	952 (21.6)	191 (33.5)	
Child body composition phenotype, n (%)			
Normal fat-normal muscle mass	3459 (78.4)	398 (69.7)	<0.001
High fat-normal muscle mass	351 (7.9)	59 (10.3)	
Normal fat-high muscle mass	265 (6.0)	37 (6.5)	
High fat-high muscle mass	338 (7.7)	77 (13.5)	

The percentages of participants with missing data on maternal age at delivery, delivery mode, birthweight, exclusive breastfeeding duration in the first six months, annual family income, maternal education, maternal current BMI, paternal education, paternal current BMI, sugary beverage consumption pattern, passive smoking in the past week, child weight status, and child body composition phenotype were 4.9%, 2.4%, 21.9%, 4.4%, 6.6%, 3.5%, 5.5%, 3.8%, 6.2%, 2.1%, 3.3%, 0.3%, and 0.3%, respectively. Abbreviation: OSA, obstructive sleep apnea; BMI, body mass index.

**Table 2:** Associations of obesity and body composition phenotype with OSA risk.

	Crude OR (95% CI)	*p*	Adjusted OR (95% CI) *	*p*
Child weight status				
Normal	1		1	
Overweight	1.57 (1.25, 1.97)	<0.001	1.53 (1.22, 1.92)	<0.001
Obesity	2.09 (1.71, 2.56)	<0.001	1.94 (1.57, 2.40)	<0.001
Abdominal obesity				
WHtR < 0.5	1		1	
WHtR ≥ 0.5	1.74 (1.45, 2.09)	<0.001	1.59 (1.31, 1.93)	<0.001
Body composition phenotype				
Normal fat -normal muscle mass	1		1	
High fat-normal muscle mass	1.46 (1.09, 1.96)	0.012	1.39 (1.03, 1.88)	0.032
Normal fat-high muscle mass	1.21 (0.85, 1.74)	0.291	1.21 (0.84, 1.73)	0.314
High fat-high muscle mass	1.98 (1.51, 2.59)	<0.001	2.05 (1.56, 2.69)	<0.001

* Multivariate models were adjusted for maternal age at delivery, maternal history of gestational diabetes mellitus/hypertension, delivery mode, child sex, exclusive breastfeeding duration in the first six months, family history of metabolic abnormalities, annual family income, maternal education, paternal education, child age, sugary beverage consumption pattern, and passive smoking in the past week. Abbreviation: OSA, obstructive sleep apnea; WHtR, waist-to-height ratio.

**Table 3:** Associations of OSA risk with metabolic abnormalities.

Metabolic Abnormalities	Crude OR (95% CI)	*p*	Model 1		Model 2	
			Adjusted OR (95% CI)	*p*	Adjusted OR (95% CI)	*p*
High TC levels	1.22 (0.89, 1.67)	0.222	1.22 (0.88, 1.68)	0.228	1.17 (0.85, 1.61)	0.339
High LDL-C levels	1.31 (1.02, 1.68)	0.036	1.27 (0.99, 1.64)	0.063	1.11 (0.86, 1.44)	0.425
Low HDL-C levels	1.41 (1.04, 1.91)	0.028	1.37 (1.01, 1.88)	0.045	1.11 (0.80, 1.54)	0.546
High TG levels	1.38 (1.09, 1.75)	0.007	1.34 (1.06, 1.71)	0.015	1.08 (0.84, 1.40)	0.531
IFG	0.89 (0.56, 1.43)	0.642	0.81 (0.50, 1.31)	0.396	0.77 (0.48, 1.25)	0.287
High UA levels	1.13 (0.93, 1.36)	0.216	1.13 (0.92, 1.37)	0.245	0.88 (0.70, 1.09)	0.240
Hypertension	1.08 (0.84, 1.39)	0.568	1.05 (0.81, 1.36)	0.704	0.79 (0.59, 1.04)	0.091

Model 1: adjusted for maternal age at delivery, maternal history of gestational diabetes mellitus/hypertension, delivery mode, child sex, exclusive breastfeeding duration in the first six months, family history of metabolic abnormalities, annual family income, maternal education, paternal education, child age, sugary beverage consumption pattern, and passive smoking in the past week; model 2: further adjusted for child BMI. Abbreviation: OSA, obstructive sleep apnea; TC, total cholesterol; LDL-C, low-density lipoprotein cholesterol; HDL-C, high-density lipoprotein cholesterol; TG, triglycerides; IFG, impaired fasting glucose; UA, uric acid.

## Data Availability

The dataset is available upon reasonable request to the corresponding author. The data are not publicly available due to privacy.

## Supplementary Materials

The following supporting information can be downloaded at https://www.mdpi.com/article/10.3390/nu16152419/s1, Table S1: Associations of OSA risk with metabolic abnormalities, stratified by the child BMI; Table S2: Associations of OSA risk with metabolic abnormalities, stratified by child sex; Table S3: Associations of OSA risk with metabolic indices.
