# Supplementary material for: Associations of Obstructive Sleep Apnea Risk with Obesity, Body Composition and Metabolic Abnormalities in School-Aged Children and Adolescents
Source: Nutrients. 2024 Jul 25;16(15):2419. doi: 10.3390/nu16152419 (PMC11313962; doi:10.3390/nu16152419)
Supplement: Supplementary file 1 [file nutrients-16-02419-s001.zip › Table S2.pdf]

**Table S2. Associations of OSA risk with metabolic abnormalities, stratified by child sex**

| | Crude OR (95%CI) | *P* | Model 1 | | Model 2 | |
|---|---|---|---|---|---|---|
| | | | Adjusted OR (95%CI) | *P* | Adjusted OR (95%CI) | *P* |
| **Boys** | | | | | | |
| High TC levels | 1.13 (0.74, 1.73) | 0.584 | 1.11 (0.72, 1.71) | 0.648 | 1.02 (0.66, 1.59) | 0.920 |
| High LDL-C levels | 1.42 (1.03, 1.94) | 0.032 | 1.40 (1.01, 1.93) | 0.044 | 1.19 (0.85, 1.67) | 0.308 |
| Low HDL-C levels | 1.22 (0.83, 1.79) | 0.318 | 1.35 (0.90, 2.01) | 0.149 | 1.14 (0.74, 1.73) | 0.554 |
| High TG levels | 1.32 (0.97, 1.80) | 0.075 | 1.30 (0.95, 1.77) | 0.103 | 1.06 (0.76, 1.47) | 0.749 |
| IFG | 0.81 (0.46, 1.43) | 0.467 | 0.82 (0.46, 1.47) | 0.506 | 0.79 (0.44, 1.42) | 0.430 |
| High UA levels | 1.01 (0.78, 1.29) | 0.963 | 1.15 (0.87, 1.53) | 0.325 | 0.90 (0.66, 1.23) | 0.498 |
| Hypertension | 1.03 (0.74, 1.42) | 0.867 | 1.05 (0.75, 1.46) | 0.788 | 0.80 (0.56, 1.15) | 0.228 |
| **Girls** | | | | | | |
| High TC levels | 1.36 (0.85, 2.18) | 0.195 | 1.36 (0.85, 2.2) | 0.205 | 1.34 (0.83, 2.17) | 0.227 |
| High LDL-C levels | 1.13 (0.75, 1.7) | 0.568 | 1.15 (0.76, 1.75) | 0.506 | 1.04 (0.68, 1.60) | 0.848 |
| Low HDL-C levels | 1.63 (1.00, 2.67) | 0.052 | 1.58 (0.95, 2.61) | 0.077 | 1.15 (0.67, 1.99) | 0.617 |
| High TG levels | 1.45 (1.00, 2.09) | 0.048 | 1.42 (0.98, 2.06) | 0.066 | 1.10 (0.74, 1.65) | 0.632 |
| IFG | 0.93 (0.40, 2.17) | 0.864 | 0.83 (0.35, 1.97) | 0.670 | 0.75 (0.32, 1.79) | 0.520 |
| High UA levels | 1.31 (0.98, 1.75) | 0.074 | 1.27 (0.94, 1.71) | 0.115 | 0.98 (0.70, 1.37) | 0.895 |
| Hypertension | 1.10 (0.73, 1.66) | 0.647 | 1.09 (0.72, 1.66) | 0.676 | 0.78 (0.49, 1.24) | 0.293 |

Model 1: adjusted for maternal age at delivery, maternal history of gestational diabetes mellitus/hypertension, delivery mode, exclusive breastfeeding duration in the first six months, family history of metabolic abnormalities, annual family income, maternal education, paternal education, child age, sugary beverage consumption pattern, and passive smoking in the past week; model 2: further adjusted for child BMI. Abbreviation: OSA, obstructive sleep apnea; TC, total cholesterol; LDL-C, low-density lipoprotein cholesterol; HDL-C, high-density lipoprotein cholesterol; TG, triglycerides; IFG, impaired fasting glucose; UA, uric acid.
