# Supplementary material for: Associations of Obstructive Sleep Apnea Risk with Obesity, Body Composition and Metabolic Abnormalities in School-Aged Children and Adolescents
Source: Nutrients. 2024 Jul 25;16(15):2419. doi: 10.3390/nu16152419 (PMC11313962; doi:10.3390/nu16152419)
Supplement: Supplementary file 1 [file nutrients-16-02419-s001.zip › Table S3.pdf]

Table S3. Associations of OSA risk with metabolic indices

Metabolic indices	Crude *β* Coefficient (95%CI)	*P*	Model 1		Model 2	
			Adjusted *β* Coefficient (95%CI)	*P*	Adjusted *β* Coefficient (95%CI)	*P*
TC	0.003 (−0.012, 0.017)	0.726	0.004 (−0.011, 0.018)	0.596	0.000 (−0.015, 0.014)	0.992
LDL-C	0.021 (−0.003, 0.045)	0.084	0.021 (−0.003, 0.046)	0.083	0.001 (−0.022, 0.025)	0.925
HDL-C	−0.036 (−0.056, −0.016)	<0.001	−0.032 (−0.051, −0.013)	0.001	−0.008 (0.026, 0.009)	0.355
TG	0.077 (0.038, 0.115)	<0.001	0.076 (0.039, 0.114)	<0.001	0.024 (−0.011, 0.058)	0.173
FPG	0.000 (−0.008, 0.009)	0.941	−0.003 (−0.011, 0.006)	0.544	−0.005 (−0.013, 0.003)	0.255
UA	0.030 (0.007, 0.052)	0.009	0.014 (−0.007, 0.034)	0.193	−0.015 (−0.033, 0.004)	0.122
SBP	0.008 (−0.001, 0.017)	0.090	0.002 (−0.006, 0.011)	0.577	−0.011 (−0.018, −0.004)	0.003
DBP	0.002 (−0.009, 0.013)	0.673	0.002 (−0.008, 0.012)	0.697	−0.008 (−0.018, 0.001)	0.095

Model 1: adjusted for maternal age at delivery, maternal history of gestational diabetes mellitus/hypertension, delivery mode, child sex, exclusive breastfeeding duration in the first six months, family history of metabolic abnormalities, annual family income, maternal education, paternal education, child age, sugary beverage consumption pattern, and passive smoking in the past week; model 2: further adjusted for child BMI. Abbreviation: OSA, obstructive sleep apnea; TC, total cholesterol; LDL-C, low-density lipoprotein cholesterol; HDL-C, high-density lipoprotein cholesterol; TG, triglycerides; IFG, impaired fasting glucose; UA, uric acid; SBP, systolic blood pressure; DBP, diastolic blood pressure.
